# Spatial–temporal evolution characteristics of land use and habitat quality in Shandong Province, China

**DOI:** 10.1038/s41598-022-19493-x

**Published:** 2022-09-14

**Authors:** Huiling Zheng, Hao Li

**Affiliations:** 1 College of Marine and Earth Sciences, Ocean University of China, Qingdao, 266100 Shandong China; 2 Center for Atmospheric Chemistry Study, Shanghai Key Laboratory of Atmospheric Particle Pollution and Prevention (LAP3), Department of Environmental Science and Engineering, Fudan University, Shanghai, 200433 China

**Keywords:** Biodiversity, Biogeography, Urban ecology

## Abstract

To explore the sustainable mechanism of land use and habitat quality, the present study examined the land cover data of Shandong Province from 1980 to 2020 to understand the spatial–temporal evolution characteristics of land use. The “Integrated Valuation of Environmental Services and Trade-off” (InVEST-HQ) model and spatial auto-correlation model were further employed to evaluate the habitat quality and analyze the relationship between its spatial distribution pattern and land use type. Our results suggested that cultivated land was the dominant land use type in Shandong Province from 1980 to 2020. During this period, the area of water and URL (urban and rural industrial and mining residential land) were gradually increased, while other land types decreased progressively. Political and socio-economic factors were the dominant factors for the evolution of land use types, which exhibited significant stage variation characteristics, and the most drastic change was observed from 2010 to 2020. We further found that habitat quality in Shandong Province was dominated by moderate degradation, whose degree of degradation was positively correlated with the degree of land use development. Moreover, the average habitat quality decreased obviously over the past 40 years, and the fastest decreased period was similar to the phase change characteristics of land use types. In addition, habitat quality was significantly clustered in spatial distribution. Hot spots (high-value areas) were mainly natural ecosystems, while cold spots (low-value areas) were mainly ecosystems that were significantly affected by human activities, such as cultivated land and URL. Our findings suggest that administrators should formulate differentiation policies, solve the development dilemma of low-level habitat quality areas and build land space security pattern to promote the ecological quality.

## Introduction

Habitat quality refers to the ability of the ecosystem to provide appropriate conditions for individual and population persistence^1,2^, playing an essential role in indicating the effectiveness of the biodiversity conservation status^3,4^. Urbanization is the most powerful and visible anthropogenic driver affecting regional habitats and biodiversity^5,6^. This process directly transforms natural land into artificial land, with a large population movement from rural to urban areas, causing environmental pollution and depletion of natural resources. It also threatens the normal functioning of natural ecosystems and affects the quantity, pattern and quality of natural habitats^7^. As the carrier of various surface ecosystems, the change of land use structure and intensity has a profound impact on the exchange process of material flow and energy flow between habitat patches. Meanwhile, this process would affect the ecosystem service function and change the regional habitat quality level^8,9^. With the rapid urbanization process, the problems related to the evolution of land use structure and habitat quality have drawn the attention at home and abroad^10^.

Habitat quality, as an indicator of regional biodiversity and the ecosystem service level, early studies on habitats mainly focused on the habitat situation of specific species and the impact of habitats on species^11,12^. For example, Katharina et al.^13^, Xu^14^, Sun^15^ and Tang et al.^16^ evaluated the habitat quality of water in Bavaria, Germany, the Yellow River Delta wetlands, and the eastern waters of Ningde with the help of biological indices. Biological index required data obtained through field investigation and collection, which could reflect the spatial–temporal changes of habitat quality more objectively and carefully. However, it tends to be studied at small geographical scales or a single species, which showed bad performance on evaluate the ecological environment at meso- and macro-scale. Moreover, with the increased impact of urbanization and industrialization on biodiversity, habitat threats and their spatial distribution have received more attention when assessing the habitat quality^17^. To address these expectations, the “habitat quality” module in the “Integrated Valuation of Environmental Services and Trade-off” model (InVEST-HQ) is gradually gaining widespread usage in assessing habitat quality by combining habitat suitability and anthropogenic threats to biodiversity, which exhibited significant advantages in terms of convenience of parameter acquisition, accuracy of result analysis, and accessibility to habitat quality assessment at different spatial and temporal scale^2,18–21^.

In recent years, how land use changes affect habitat quality have been widely reported^22,23^. For example, urban expansion significantly disrupted the natural habitat, leading to habitat fragmentation and degradation^24–26^. Besides, natural environment (topography, climate) and land use type also significantly influence the change of habitat quality^27–29^. The influences of such natural factors are relatively fixed, while land use categories are affected by a wide range of human activities. It is generally believed that habitat quality is low in plain areas and high in hilly and mountainous areas^30^, which is mainly because the high-altitude areas are difficult to develop and rarely affected. However, with the progress of society, high-altitude mountainous and hilly areas have also been widely exploited, the spatial distribution pattern of habitat quality has changed significantly. In view of the differences in the natural environment and the degree of exploitation, the spatial pattern and evolution of habitat quality need to be analyzed according to local conditions and specific problems. What’s more, habitat quality is characterized by spatial and temporal dynamics^7,31^, therefore, long-series data was more accurately to evaluate the evolution characteristics of regional habitat quality in a comprehensive manner^32–35^.

As the second most populous province and the third most economical province in China, Shandong Province has experienced rapid socio-economic development and urbanization since China implemented the reform and opening-up policy in 1978. Drastic changes have taken place in various land types, and the habitat quality issues have come to the fore. In recent years, with the acceleration construction of the Shandong Peninsula Blue Economic Zone and the Yellow River Delta Efficient Ecological Economic Zone, Shandong’s economic development has entered a new stage. The contradiction between economic development and ecological protection needs urgent attention. At present, Huang^36^ analyzed the habitat quality pattern in Shandong Province based on land use data for1980, 1995, 2005, and 2015. However, constrained by the timeliness and representativeness, the study just focused on the spatial distribution of habitat quality. Sun et al.^37^ only analyzed the trend of habitat quality in Shandong Province since the 21st century. Long-term land use patterns and the evolution characteristics of habitat quality have not been revealed. Meanwhile, the relationship between land use and habitat quality in Shandong Province are still poorly understood. Therefore, we explore the evolution pattern of regional habitat quality by analyzing the land use change of Shandong Province in the past 40 years (1980–2020) with the InVEST-HQ model and GIS, which was conducted to pinpoint the characteristics of spatial–temporal evolution and phase change of land use in Shandong Province from 1980 to 2020. Furthermore, the spatial–temporal variation characteristics and the relationship between spatial distribution and land use type were revealed in the region. At last, we summarize suggestions to optimize regional land use pattern and improve ecological environment, and provide reference information for regional territorial spatial planning and ecological civilization construction.

## Materials and methods

### Study area

**Figure 1:**
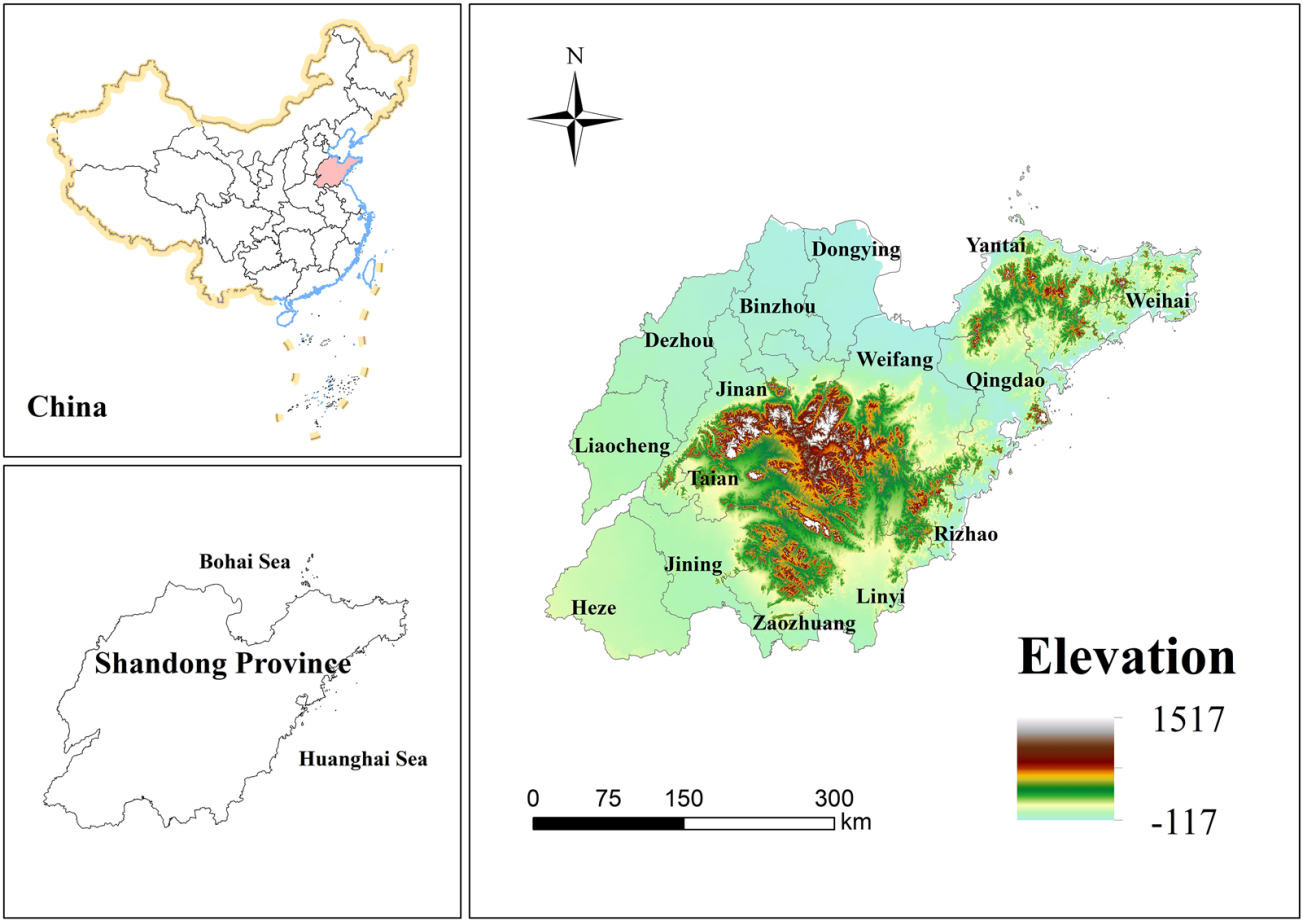
Study area.

Shandong Province is located on the eastern coast of China (34°22.9′–38°24.01′N, 114°47.5′–122°42.3′E) (Fig. 1). The Shandong Peninsula stands out from the Bohai Sea and the Huanghai Sea, and faces the Liaodong Peninsula at a distance. The inland part borders Hebei Province, Henan Province, Anhui Province, and Jiangsu Province. Shandong Province is a mountainous region in the central part of the country, with low-lying and flat areas in the southwest and northwest, and gently rolling hills in the east. The overall landform includes the northwest plain of Shandong, the Central and South Shandong Mountains and the Jiaodong Hills. The climate in the province belongs to the warm temperate monsoon climate, with concentrated precipitation and the same period of rain and heat. The annual average temperature is 11–14 °C. There are sufficient light resources with the annual mean light hours of 2290–2890 h. The heat conditions can meet the needs of crops twice a year. The complex topography and climate have caused a diversity of land use type. As a populous and economic province in eastern China, the urbanization rate increased from 13.19% in 1985 to 61.51% in 2020. Rapid economic development and urban expansion have led to dramatic changes in regional land use type, resulting in further destruction and increased fragmentation degree of regional habitat, as well as significant changes in habitat quality.

### Data sources

In this study, land use raster datasets (1 km*1 km) of Shandong Province in 1980, 1990, 2000, 2010 and 2020 were derived from the Resource and Environment Science and Data Center (http://www.resdc.cn), which was classified into 7 primary land types (Class I) and 24 secondary land types (Class II), as shown in Table 1.

**Table 1:** Land use classification system in the study area.

Class I	Class II	Code	Class I	Class II	Code	Class I	Class II	Code
Cultivated land (CL)	Paddy field	11		Low coverage grassland	33		Other construction land	53
	Dry land	12	Water area (WA)	River	41	Unused land (UL)	Sandland	61
Forest (F)	Woodland	21		Lake	42		Saline alkali land	63
	Shrubwood	22		Reservoir pit	43		Marshland	64
	Sparse wood	23		Mudflat	45		Bare land	65
	Other woodland	24		Beach	46		Bare rock land	66
Grassland (GL)	High coverage grassland	31	Urban and rural industrial and mining residential land (URL)	Urban land	51		Other unused land	67
	Medium coverage grassland	32		Rural settlement	52	9	Ocean (O)	99

## Methods

### Land use change analysis

We used the spatial overlay function of ArcGIS10.2 for land cover data from 1980 to 2020 to obtain the land use characteristics, conversion direction and transferred area of Shandong Province at different periods.

### Habitat quality evaluation

The InVEST-HQ model was used to assess habitat quality, which would reflect the impact of human activities on environment. High intensity human activities can threaten ecosystems and lead to poor habitat quality and vice versa. The data of land use and threat factor was used through relevant equations to analyze the impact of threat sources on habitat^38^.

Habitat quality module include habitat degradation degree and habitat quality evaluation. Habitat degradation degree was calculated by the following formula.

1 $${D}_{xj}=\sum_{r=1}^{r}\sum_{y=1}^{y}\left(\frac{{w}_{r}}{\sum_{n=1}^{n}{w}_{r}}\right){r}_{y}{i}_{rxy}{\beta }_{x}{S}_{jr}$$

2 $${i}_{rxy}=1-\left(\frac{{d}_{xy}}{{d}_{rmax}}\right) \;\;(\mathrm{linear})$$

3 $${i}_{rxy}=exp\left(-\frac{2.99{d}_{xy}}{{d}_{rmax}}\right)\;\;(\mathrm{exponential})$$

where *D*_*xj*_ denotes the total threat level in grid cell *x* with land type *j*, *r* denotes the number of threat factors, *y* indicates the set of grid cells on *r*’s raster map, *w*_*r*_ is the impact weight of threat *r*, *i*_*rxy*_ means the degradation decay function through distance, which can be expressed as the linear or exponential function of distance from threats to habitats, *β*_*x*_ denotes the accessible grid cell *x*; *S*_*jr*_ indicates the relative sensitivity of land type *j* to threat factor *r, d*_*xy*_ denotes the distance between pixel *x* and pixel *y*; *d*_*rmax*_ is the maximum impact distance of threat of *r* originated in pixel *y.* The value of habitat degradation ranged from 0 to 1, and the greater the value, the more obvious the degradation^38^.

Habitat quality was calculated by the following formula.

4 $${Q}_{xj}={H}_{j}\left(1-\frac{{D}_{xj}^{2}}{{D}_{xj}^{2}+{k}^{2}}\right)$$

where *Q*_*xj*_ denotes the habitat quality of raster *x* with land type *j*, *H*_*j*_ is the habitat suitability of land type *j*, *k* is the half saturation constant. The value of habitat quality ranged from 0 to 1, with higher value indicating better habitat quality^34^.

The present study selected cultivated land, URL and unused land as threat factors according to research status, existing relevant studies^39–43^ and expert opinions. We set three threat factor parameters (Table 2) and sensitivity of habitat types to each threat factor parameters (Table 3).

**Table 2:** The threat factors and related coefficients.

Threat factor	*d*_*rma*_ (km)	Weight *w*_*r*_	Distance–decay function
Cultivated land	3	0.6	Linear
Urban land	10	0.8	Exponential
Rural settlement	5	0.6	Exponential
Other construction land	8	0.7	Exponential
Unused land	1	0.5	Linear

**Table 3:** Sensitivity of habitat types to each threat factor.

Habitat type	Habitat suitability	Threat factor				
		Cultivated land	Urban land	Rural settlement	Other construction land	Unused land
Paddy field	0.6	0.3	0.5	0.35	0.3	0.2
Dry land	0.5	0.3	0.5	0.35	0.5	0.2
Woodland	1	0.7	0.9	0.8	0.8	0.5
Shrubwood	1	0.6	0.8	0.6	0.7	0.4
Sparse wood	1	0.7	0.8	0.7	0.8	0.5
Other woodland	1	0.7	0.8	0.7	0.8	0.4
High coverage grassland	0.9	0.4	0.5	0.5	0.4	0.4
Medium coverage grassland	0.8	0.5	0.6	0.55	0.6	0.45
Low coverage grassland	0.7	0.6	0.7	0.65	0.65	0.5
River	1	0.65	0.85	0.7	0.45	0.4
Lake	1	0.7	0.9	0.75	0.5	0.4
Reservoir pit	1	0.7	0.9	0.75	0.5	0.4
Mudflat	0.6	0.6	0.8	0.8	0.55	0.5
Beach	0.6	0.6	0.8	0.8	0.55	0.5
Urban land	0	0	0	0	0	0
Rural settlement	0	0	0	0	0	0
Other construction land	0	0	0	0	0	0
Sandland	0	0	0	0	0	0
Saline alkali land	0	0.1	0.3	0.3	0.2	0
Marshland	0.3	0.6	0.2	0.3	0.2	0
Bare land	0.2	0	0	0	0	0
Bare rock land	0	0	0	0	0	0
Other unused land	0.2	0.1	0.4	0.3	0.3	0
Ocean	1	0.3	0.5	0.2	0.3	0

### Spatial auto-correlation analysis

Spatial auto-correlation captures the possibility that the values of spatial variables at nearby locations are similar^44^. In this study, the global Moran’s I index and local Getis-Ord G^⁎^ index were used to reflect cluster characteristics of habitat quality. Values of Moran’s I range from − 1 to 1 after variance normalization and represents different meanings of spatial correlation.

i. Moran’s I > 0, indicates a positive spatial correlation and perfect clustering of similar values.
ii. Moran’s I = 0, indicates the space is no autocorrelation (perfect randomness).
iii. Moran’s I < 0, indicates a negative spatial correlation and clustering of dissimilar values.

Local auto-correlation Getis-Ord G⁎ index is judged by the size of Z-score. Z > 0, indicates the clustering of high value of habitat quality (forming hot spot). Z < 0, indicates the clustering of low value of habitat quality (forming cold spot).

## Results and discussion

### Spatial–temporal characteristics of land use change

**Figure 2:**
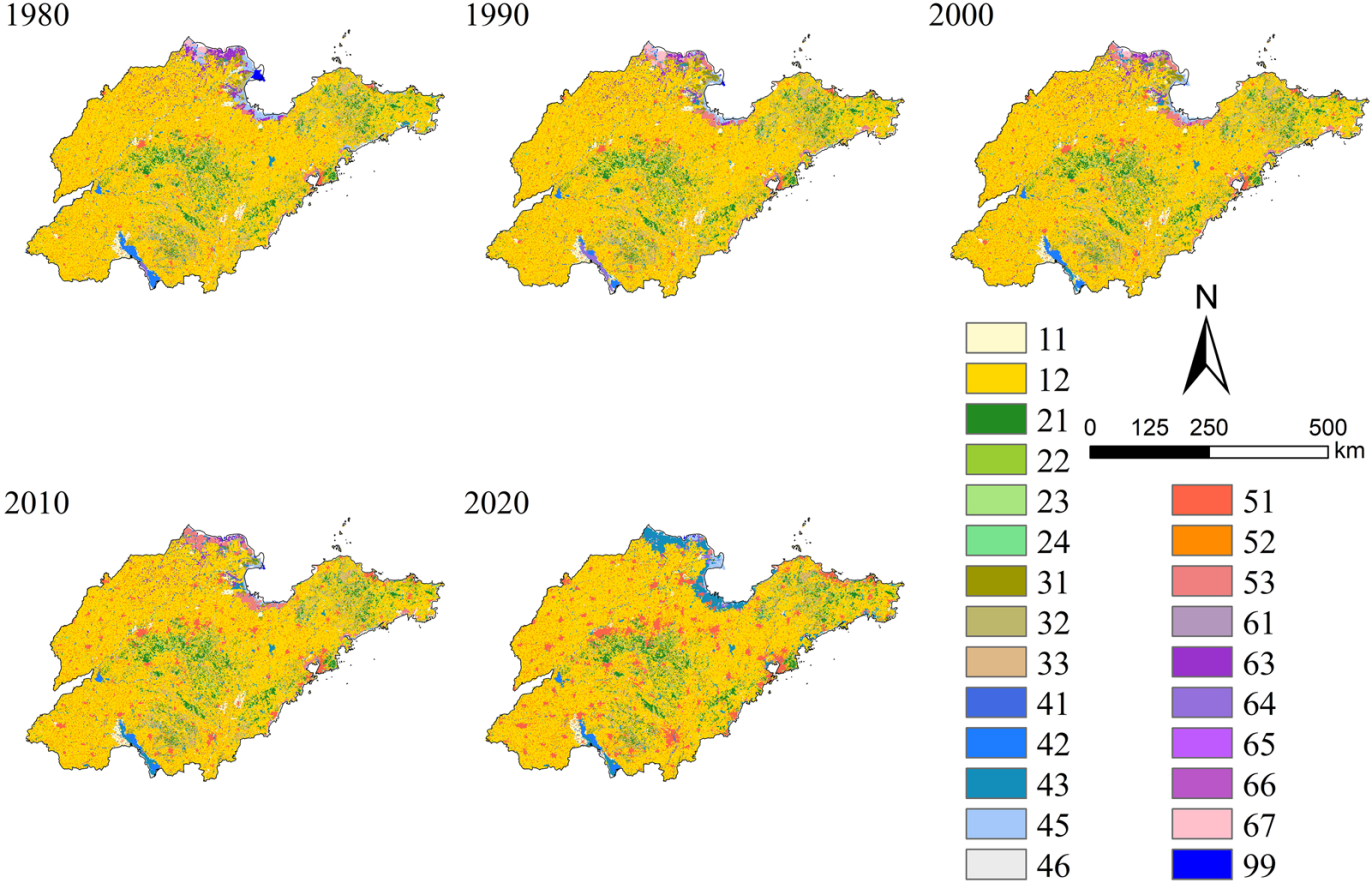
Land use type map of Shandong Province from 1980 to 2020.

**Figure 3:**
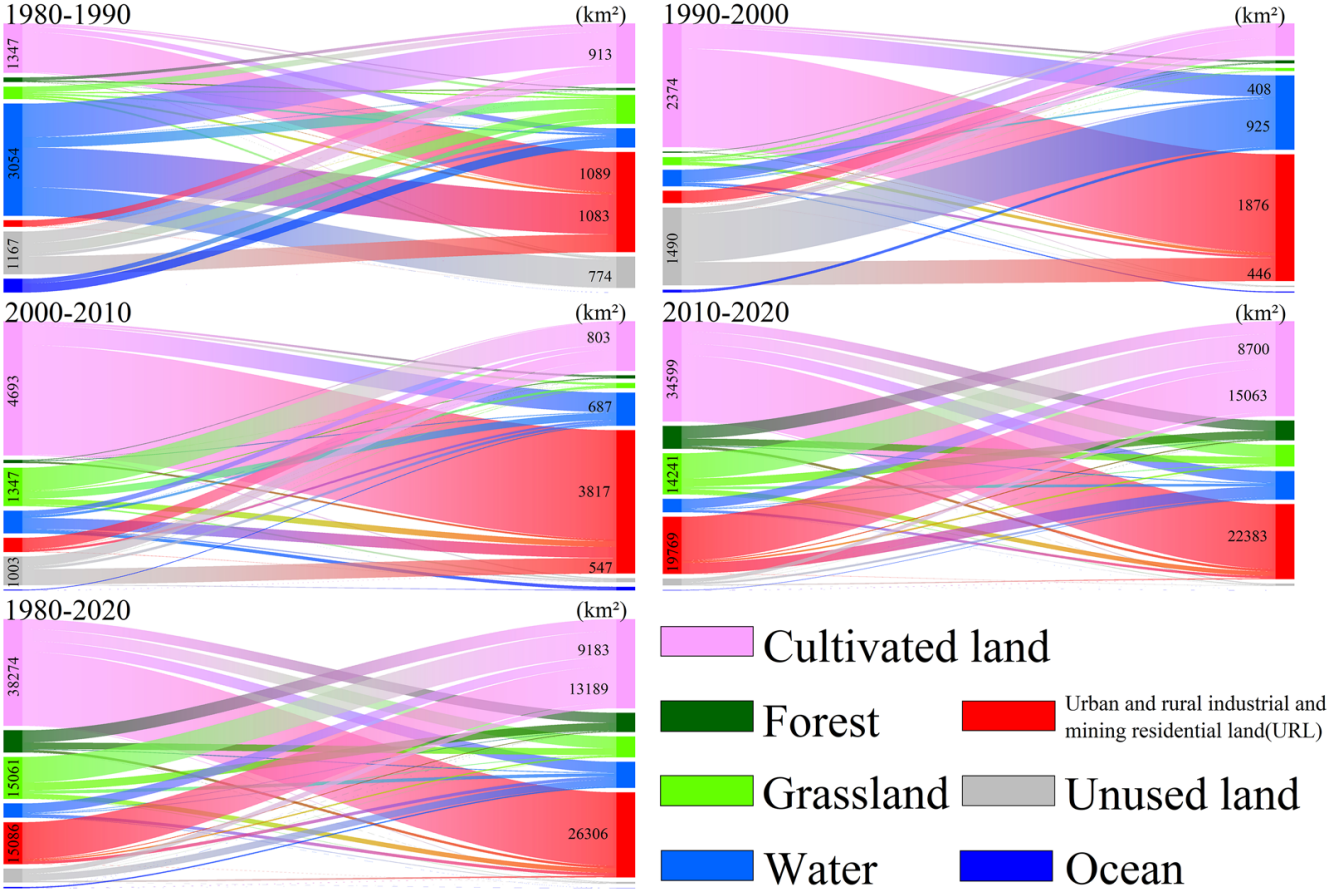
Sankey diagram of land use transfer in different periods.

As shown in Fig. 2, cultivated land was the dominant land use type in Shandong Province during the past 40 years, which accounted for 69.86% (1980), 69.98% (1990), 69.25% (2000), 68.00% (2010) and 66.88% (2020) respectively. Moreover, it was found that the area of cultivated land, forest land, grassland, unused land and ocean gradually decreased, whereas the water area and URL (urban and rural industrial and mining residential land) increased obviously. In particular, grassland decreased by 7542.87 km^2^ in the past 40 years with a decline rate of 37.18%, which was much higher than cultivated land and forest land. This phenomenon was attributed to the fact that cultivated land and forest land were less susceptible to encroachment as their high vegetation coverage, while grassland was easily occupied by other land types. The serious occupation by other land types has led to a significant reduction in unused land with a very high decline ratio of 64.32% from 2010 to 2020. In contrast to unused land, URL increased significantly at this period (Fig. 3), which was due to the rapidly economic development.

The total area of land use conversion in Shandong Province was 86,909 km^2^ during the past 40 years, the most drastic change was observed from 2010 to 2020. On the one hand, the major project of new and old kinetic energy conversion in Shandong Province had been implemented since 2000, which led to the expansion of urban land and dramatic changes in land use patterns. On the other hand, social, economic, technological and other factors had a direct impact on land use change by influencing people’s decision-making on land use (e.g., demand for land products, investment in land, protection of land resources, etc.)^45–48^. Statistics showed that GDP (Gross Domestic Product) and population density of Shandong Province had increased significantly since 21st century. The GDP of 2010–2020 was about 10 times that of 1980–2000 and population density had also increased by 1.4 times (Data from: *Shandong statistical yearbook*, http://tjj.shandong.gov.cn/col/col6279/index.html). As the most direct reflection of human activities, land use change was obviously affected by factors such as agricultural cultivation, industrial and mining construction, and urbanization driven by population growth^49,50^.

**Figure 4:**
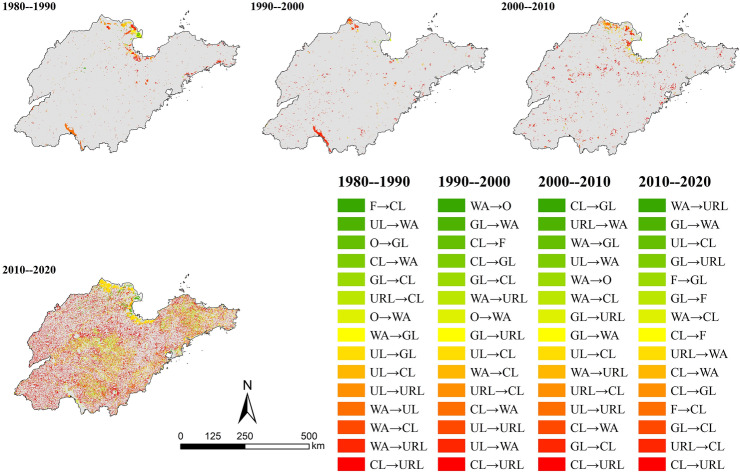
Spatial distribution map of land use conversion types in different periods.

The most significant changes of land use type were URL (increased by 17.75%), grassland (decreased by 8.72%) and cultivated land (decreased by 7.26%) over the past forty years. URL was mostly converted from cultivated land (26,306 km^2^) and grassland (1684 km^2^), which reflected the serious situation of occupying cultivated land in the process of urbanization in Shandong Province. It was caused by tight land use scale and relatively flat terrain of grassland. Besides, the range of land use type in the four periods also exhibited great variations. The conversion of land use from 1980 to 1990 was concentrated in the Yellow River Delta, Laizhou Bay and Weishan Lake, for the same as 1990–2000. At the period of 2000 to 2010, the conversion types concentrated in Bohai Bay and Yellow River Delta. The land use conversion was violent and widely distributed from 2010 to 2020, which was different from previous periods from 1980 to 2010. The conversion of cultivated land → URL and URL → cultivated land were widely distributed in Shandong Province, while another conversion of grassland → cultivated land and forest → cultivated land were concentrated in the Central and South Shandong Mountains and Jiaodong Hills. In addition, the conversion of cultivated land → water area and URL → water area were concentrated in Bohai Bay, Yellow River Delta and Laizhou Bay. Ample water, flat terrain and fertile soils in these bays and deltas facilitates agricultural cultivation and other productive activities. Therefore, the conversion of land use types from 1980 to 2010 was mainly concentrated here (Fig. 4). Specifically, the conversion of water area → URL was 1083 km^2^ from 1980 to 1990, unused land → water area was 925 km^2^ from 1990 to 2000, cultivated land → water area was 687 km^2^ from 2000 to 2010. However, the pattern of land use change dominated by natural factors has been broken in the process of increasing demand for social development and continuous advancement of science and technology. The conversion of land use types has become more dispersed in spatial distribution and the types of conversion have become more diverse.

In fact, one issue of concern in the early exploitation of water was the ecological problems caused by over-exploitation. For example, the cut-off of the Yellow River downstream made it difficult to guarantee the water security of industrial and agricultural production and residential life in the areas along the way. At the same time, the safety of coastal ecosystems was threatened and the phenomenon of soil salinization had become more serious. To alleviate these problems, government and the public have taken a series of measures such as establishing the Yellow River Delta National Nature Reserve was established in 1992, returning farmland to lakes and wetlands, and improving the landscape pattern of rivers and lakes by carrying out ecological treatment in the coastal zone of rivers and lakes^51,52^. By 2020, the area of water has increased by 50% compared to 1980, while many ecological security issues have been mitigated.

### Spatial–temporal characteristics of habitat degradation

The spatial–temporal variation of land use types were conducted to explore the variation trend of its habitat quality in Shandong Province. The InVEST-HQ was applied to obtain layers of habitat degradation in different periods. According to the interval range of 0–0.03, 0.03–0.07 and 0.07–0.18, habitat degradation was divided into three levels: slight, moderate and high degradation^35,38^.

**Figure 5:**
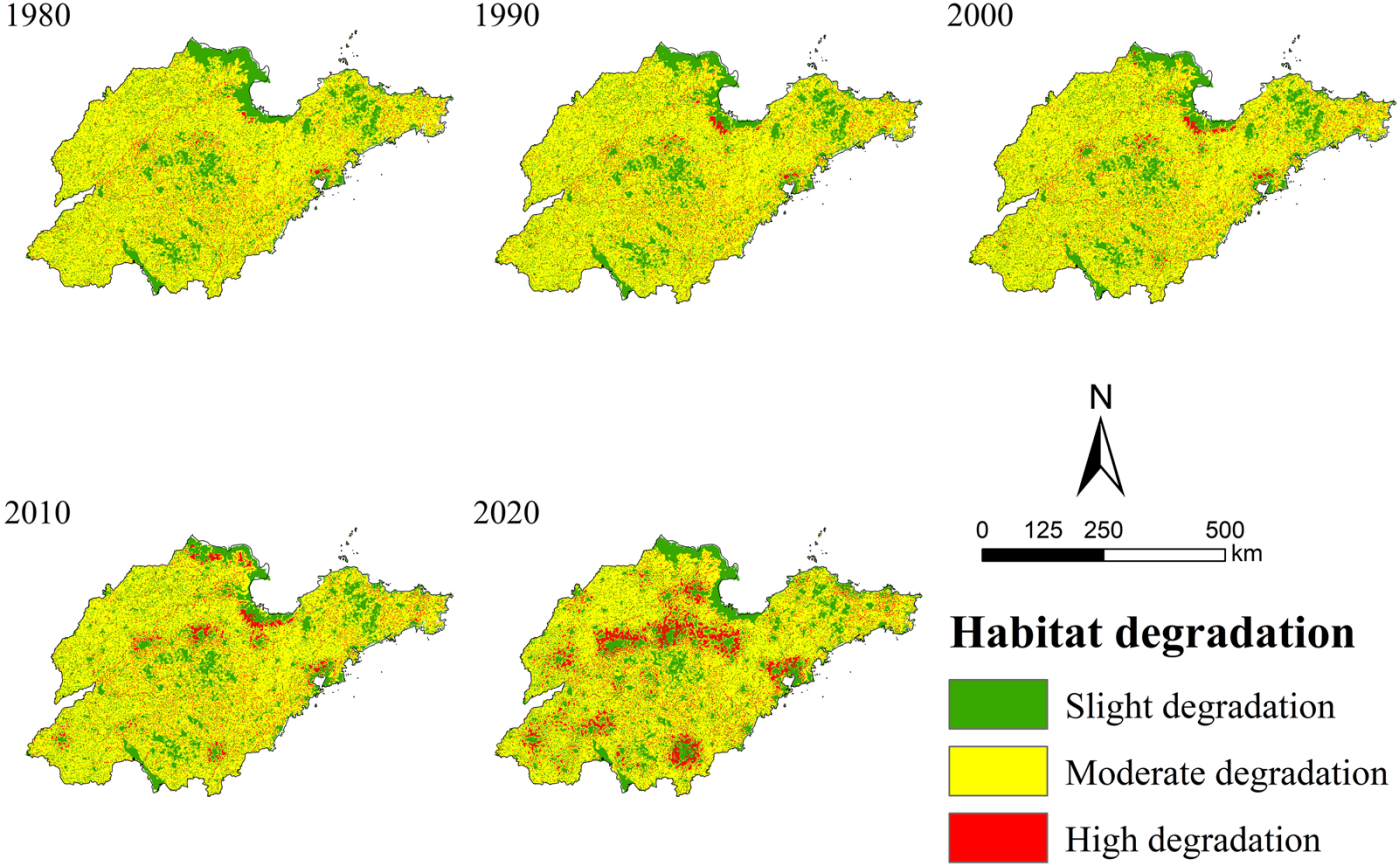
Distribution map of habitat degradation in Shandong Province from 1980 to 2020.

As shown in Fig. 5, the habitat quality in Shandong Province was dominated by moderate degradation, with the proportion of 73.30% (1980), 73.25% (1990), 72.49% (2000), 70.45% (2010) and 64.33% (2020), respectively. The spatial pattern of habitat quality was consistent with cultivated land, indicating that cultivated land who was affected by natural and anthropogenic activities exhibited moderate degradation. The proportion of moderate degradation has decreased due to cultivated land have been encroached upon for construction in the process of development, thus habitat degradation has become more and more serious. Although some of the moderate degraded areas were also converted to slight degraded areas, the area of conversion was very small compared to its conversion to high degraded areas.

The proportion of slight degradation ranges from 22.38% to 24.89%, it was concentrated in the Yellow River Delta, the Central and South of Shandong Mountains, Weishan Lake and Jiaodong Hills, which was less disturbed by human activities. Compared with 1980, the proportion of slight degraded areas increased marginally in 2020, and its change was a fluctuating process. The proportion of slight degraded areas decreased from 1980 to 1990, and its proportion slowly increased from 1990 to 2020. This dynamic change process could be verified according to the spatial distribution characteristics in the Yellow River Delta. The habitat quality of the Yellow River Delta, which originally showed slight degradation, showed high degradation in 1990, 2000 and 2010.

The proportion of high degradation ranges from 4.03% to 10.78%, which was concentrated in the built-up area of the city where human activities were more intensive. The proportion of high degraded areas has been increasing, indicating that the habitat has been degraded severely and its quality has declined. As the proportion of high degraded areas raised, two patterns of their spatial distribution also emerged. First spatial pattern was concentrated in urban built-up areas because of the high degree of human exploitation of land, which led to significant habitat degradation. The second pattern was a circle structure with “slight degradation” as the center and “high degradation-moderate degradation-slight degradation” outward, which was similar to the spatial distribution structure of habitat degradation in Fujian Province studied by Li et al.^40^. The circle structure was formed in 2010, and the distribution range was significantly expanded in 2020. The reason for the formation was that the built-up land in the city center has been severely damaged, and the possibility of re-degradation was reduced, instead showing “slight degradation”. However, the adjacent urban areas were more threatened and severely degraded, presenting “high degradation”. With the increase of distance, habitat threat and degradation decreased gradually, displaying “slight degradation”.

### Spatial–temporal evolution characteristics of habitat quality

The InVEST-HQ was used to obtain layers of habitat quality in different periods. As summarized in Table 4, habitat quality was divided into five levels by the interval range: low (0–0.2), relatively low (0.2–0.4), medium (0.4–0.6), relative high (0.6–0.8), and high (0.8–1.0)^35,38^.

**Table 4:** The proportion of habitat quality level at different periods in Shandong Province.

Level	Interval	1980		1990		2000		2010		2020	
		Grids/piece	Proportion/%	Grids/piece	Proportion/%	Grids/piece	Proportion/%	Grids/piece	Proportion/%	Grids/piece	Proportion/%
Low	[0–0.2)	30,458	12.67	32,714	13.61	34,306	14.28	37,740	15.70	42,167	17.44
Relative low	[0.2–0.4)	1103	0.46	1117	0.46	243	0.10	257	0.11	563	0.23
Medium	[0.4–0.6)	165,684	68.95	164,711	68.54	162,772	67.74	159,513	66.37	158,290	65.47
Relative high	[0.6–0.8)	15,100	6.28	15,334	6.38	15,365	6.39	14,628	6.09	9371	3.88
High	[0.8–1)	27,965	11.64	26,431	11.00	27,616	11.49	28,217	11.74	31,394	12.98

Our study concluded that the level of habitat quality in Shandong Province declined from 1980 to 2020.The results showed an overall decline of 4.75% in Shandong Province. Among them, the most significant rate of decline was observed in 2010–2020 (1.86%), which was similar to the phase change characteristics of land use types. At this period, the “Development Plan of Yellow River Delta Efficient Ecological Economic Zone” and the “Development Plan of Shandong Peninsula Blue Economic Zone” have become national development strategies. The demonstration area of “Bohai granary” and the restructuring of steel industry were carried out simultaneously. Meanwhile, the Beijing-Shanghai high-speed railway (Shandong section), Qingdao Jiaozhou Bay Bridge, Jiaozhou Bay Tunnel have strengthened the connection between Shandong Province and the outside world. As a result, rapid development has led to a rapid decline in the quality of its habitat. The rate of decline in 1980–1990 (1.43%) and 2000–2010 (1.42%) was comparable and the rate of decline in 1990–2000 was the lowest at 0.12%, which was significantly related to the development level of cities in each period. The period of 1980–1990 and 2000–2010 were in the initial and rapid promotion stages of reform and opening-up respectively. The initial stage was led by rural reform, and urban reform was launched on a pilot basis. The rapid advancement stage was led by urban reform, and economic development entered a healthy track of steady progress. Therefore, the proportion of habitat quality changes in the two periods was comparable. The period of 1990–2000 was in the exploration and transition stage of reform and opening-up, whose development process was relatively stable, resulting in the lowest rate of change in habitat quality.

**Figure 6:**
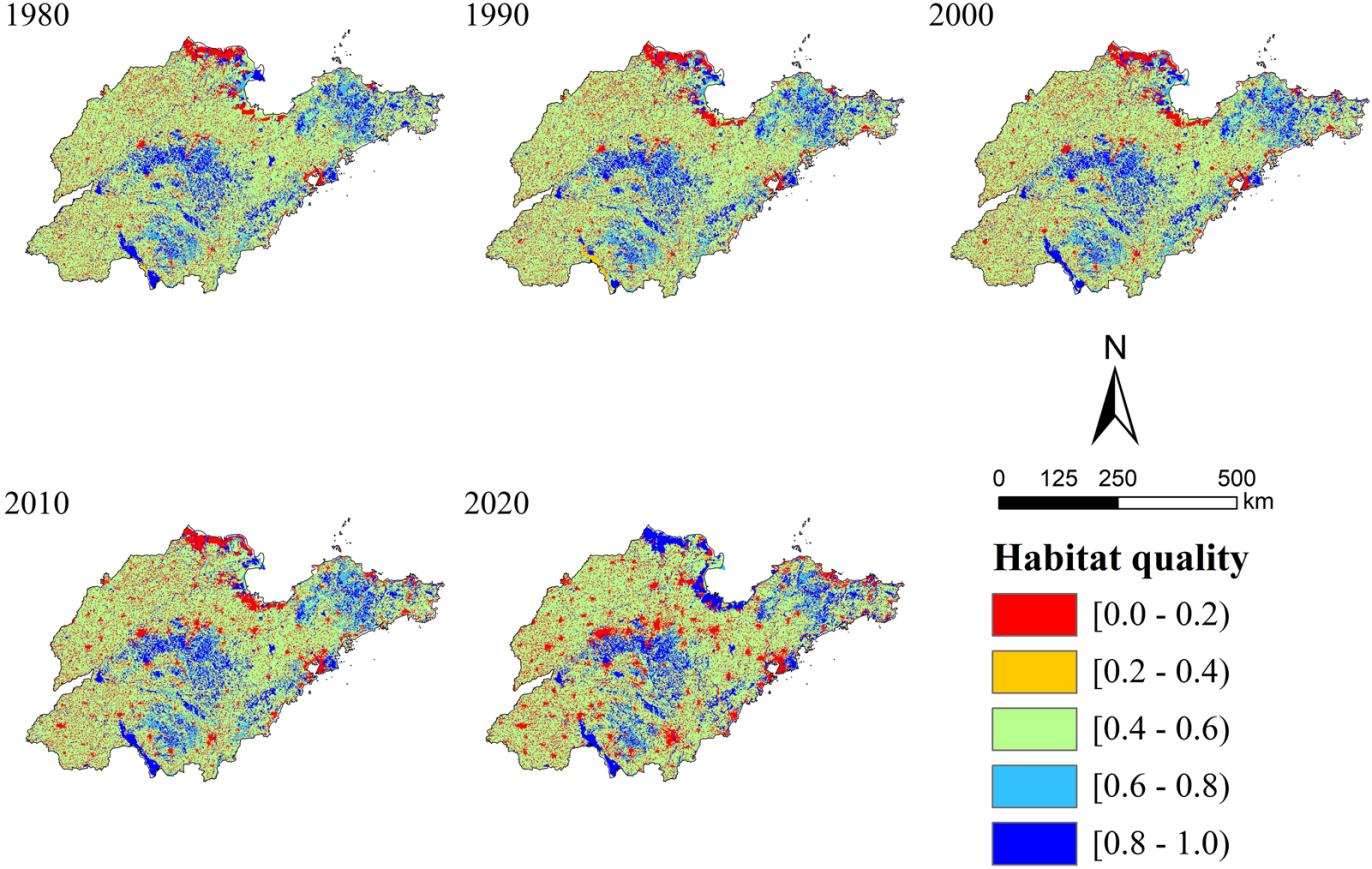
Distribution map of habitat quality in Shandong Province from 1980 to 2020.

The average value of habitat quality in Shandong Province was 1980 (0.5091), 1990 (0.5018), 2000 (0.5012), 2010 (0.4941) and 2020 (0.4849), which decreased during the entire period. Habitat quality was dominated by medium-level throughout the whole period, with the proportion in 1980 (68.95%), 1990 (68.54%), 2000 (67.74%), 2010 (66.37%) and 2020 (65.47%). The land type in this category was mainly cultivated land (Fig. 6), which was continuous encroachment during the study period, resulting in a decrease in the percentage of medium-level habitat quality. From 1980 to 2020, the percentage of low-level habitat quality increased from 12.67% to 17.44%, and the relatively low-level decreased from 0.46% to 0.23%. The main reason was the continuous increasing of construction land and the degree of habitat threat led to the decreasing of habitat suitability. Therefore, the area of low-level habitat quality showed an increasing trend. Low and relative low-level habitat quality areas were concentrated in the urban areas of coastal and inland cities, and the Yellow River Delta. Urban areas, with a large scale of industry, commerce and population, also have a high level of urbanization. The original natural habitat has been modified during the development process, which resulted low-level habitat quality. The habitat quality of the Yellow River Delta was dynamic. The low-level pattern formed by early over-exploitation was improved in later conservation and development. The proportion of high-level habitat quality increased from 11.64% to 12.98%, and the relatively high-level decreased from 6.28% to 3.88%. In terms of spatial distribution, it was concentrated in the Central and South Shandong Mountains, Jiaodong Hills, the Yellow River Delta (2020), Weishan Lake and Wulian Mountain. These areas were dominated by mountains and well-protected water, which had high habitat suitability and were less stressed by surrounding construction land, thus maintaining high-level habitat quality. The increase of high-level habitat quality was due to the influence of water with high habitat suitability, which expanded a lot in the past 40 years, leading to the spread of high-level regional habitat quality, especially in the Yellow River Delta.

The value of Moran’s I was 0.3935 (1980), 0.3852 (1990), 0.4031 (2000), 0.4186 (2010) and 0.4644 (2020), respectively, which revealed that the spatial agglomeration of habitat quality in Shandong Province was characterized by agglomeration, and the trend of agglomeration increased obviously after 2000.

**Figure 7:**
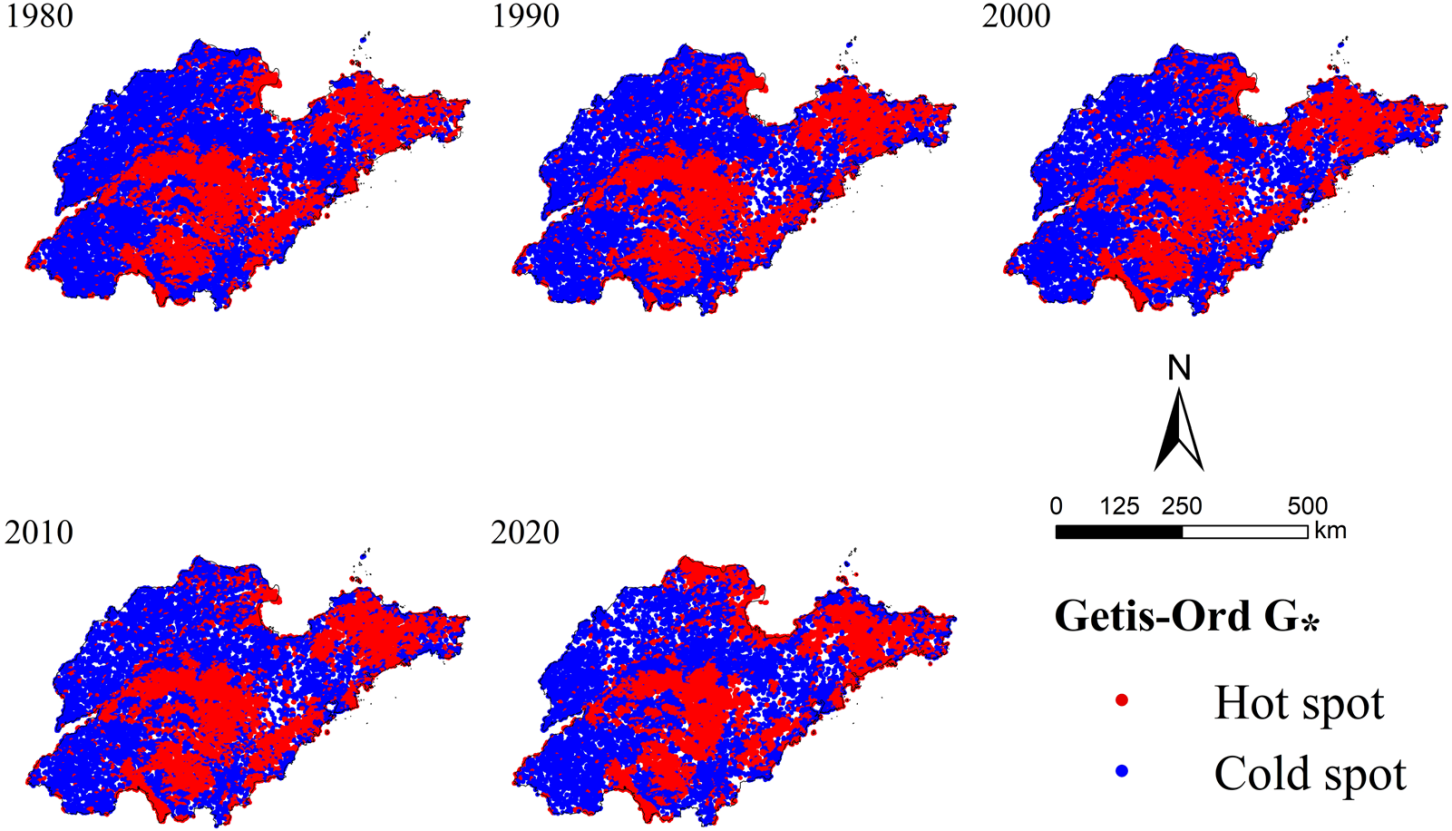
Distribution map of hot and cold spots of habitat quality in Shandong Province from 1980 to 2020.

As shown in Fig. 7, the habitat quality in Shandong Province exhibited obvious spatial heterogeneity, and spatial distribution of cold and hot spot was consistent with the topographic features. Hot spot (high-value area of habitat quality) presented “two primary and two secondary + Yellow River Delta”. Two primary hot spots distributed in the Central and South Shandong Mountains and the Jiaodong Hills, the two secondary hot spots located in Weishan Lake and Wulian Mountain. The formation of above hot spot was mainly due to high altitudes or steep slopes conferred favorable habitat quality, which was associated with the accessibility of human activities. Human accessibility at high altitudes or steep slopes was limited, so it was unlikely to cause major interference with the original environment^53,54^. However, the formation of other hot spot in Yellow River Delta was due to protective human activities. Cold spot (low-value area of habitat quality) was scattered in the northwestern Plain of Shandong Province, provincial capital metropolitan area and peninsula urban agglomeration which was dominated by cultivated land and built-up land in the cities that was affected by agricultural cultivation and industrial activities.

Overall, the spatial distribution pattern of habitat quality in Shandong Province was relatively stable and affected by many factors, among which land use change was the most important one^9,40,55^. The most dominant land type in Shandong Province was cultivated land, which was concentrated in the northwest plain. Influenced by agricultural farming, the habitat quality of cultivated land presented medium-level category. At the same time, the habitat quality of some cultivated land has decreased due to the influence of construction land intrusion. The high vegetation coverage and rich species diversity of mountains and hills make their natural habitat quality superior. With the development of urban economy, the scale of construction land in coastal lowlands as well as inland urban areas continued to expand. The increase in population density as well as the intensity of land use activities has led to the expansion of regional dehabitatization. In addition, the dynamic changes in the habitat quality of the Yellow River Delta indicated that differences in the degree of land use change led to a variety of impacts on habitat quality. Therefore, habitat quality improvement and ecological protection should be based on local regional resource endowments and follow the concept of comprehensive, coordinated and sustainable development. Administration should formulate differentiated ecological protection strategies. For urban land development, authorities should increase the intensive utilization of construction land, limit the development boundaries of urban land and increase the greening rate inside urban land, such as equipped with urban green space park and other ecological land. In order to ensure the efficiency of agricultural production in Shandong Province, authorities should pay special attention to the conservation of cultivated land and to the development of ecological agriculture^56^. For natural ecosystems such as forest and grassland, authorities should improve the natural reserve system^57^. The vegetation ecological restoration project should be carried out according to local conditions. Drawing on the effective experience of ecological changes in the Yellow River Delta, we would take it as a typical example in future development and adopt corresponding administrative methods to coordinate the relationship between economy and habitat quality and change the dilemma of low-level habitat quality areas. Therefore, it is necessary to implement reasonable and effective territorial space planning to achieve regional sustainable development.

## Conclusions

Based on 40-year of land cover data in Shandong Province, this paper evaluated its habitat quality with the InVEST-HQ model, and focused on long-term evolution characteristics of habitat quality and the relationship between its spatial distribution pattern and land use. The main conclusions are that:

1. Over the past 40 years, the land use type in Shandong Province had been dominated by cultivated land. The area of URL and water increased significantly, while other land types showed a decreasing trend. There are stage differences in land use type changes, with the most dramatic changes occurring from 2010 to 2020.
2. In general, the habitat quality in Shandong Province had been dominated by moderate degradation in the past 40 years and the degree of degradation was closely related to the land use type. Mountainous and hills, which were less affected by human activities, showed a slight degradation pattern. However, areas with intensive human activities, such as urban agglomerations, showed a high degradation pattern. And the spatial distribution of cultivated land was consistent with that of moderate degraded areas.
3. Habitat quality in Shandong Province declined significantly from 1980 to 2020, and the most significant rate of decline was observed in 2010–2020, which was similar to the phase change characteristics of land use types. What's more, the spatial distribution of habitat quality in Shandong Province was characterized by agglomeration, forming obvious hot spots and cold spots. The land use types in high-value habitat quality areas were mainly natural ecosystems such as forest and grassland, while low-value areas were mainly ecosystems that were significantly affected by human activities, such as cultivated land and URL. It reflected the complex relationship between habitat quality and land use type.

This paper revealed the spatial–temporal evolution characteristics of land use pattern and habitat quality in Shandong Province since 1980s, which could provide an empirical basis for relevant theoretical research and regional development planning. However, limited by the assessment methods of habitat quality, multi-source data and more precise and representative indicators should be used to improve the accuracy of habitat quality assessment. In addition, the threat factors should be determined by field surveys in future studies and combined with InVEST model to make the results of habitat quality evaluation more accurate.

## Supplementary Information

Supplementary Information.

## Data Availability

All data generated or analysed during this study are included in this published article [and its supplementary information files]. The land use raster datasets (1 km * 1 km) of Shandong Province in 1980, 1990, 2000, 2010 and 2020 were derived from the Resource and Environment Science and Data Center (http://www.resdc.cn). The raw data has been uploaded as supplementary files. Habitat quality index data has been uploaded as supplementary files.
