# Supplementary figures and images for: Spatial–temporal evolution characteristics of land use and habitat quality in Shandong Province, China
Source: Sci Rep. 2022 Sep 14;12:15422. doi: 10.1038/s41598-022-19493-x (PMC9475025; doi:10.1038/s41598-022-19493-x)

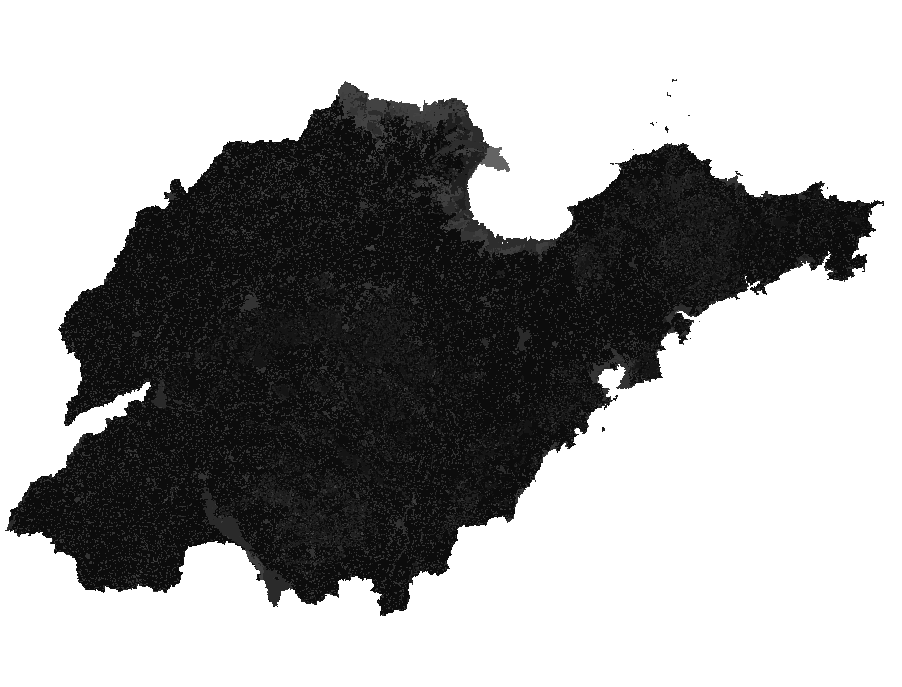

Supplement: Supplementary file 1 — Supplementary Information. [file 41598_2022_19493_MOESM1_ESM.zip › 41598_2022_19493_MOESM1_ESM/Data-tif/1980.tif]

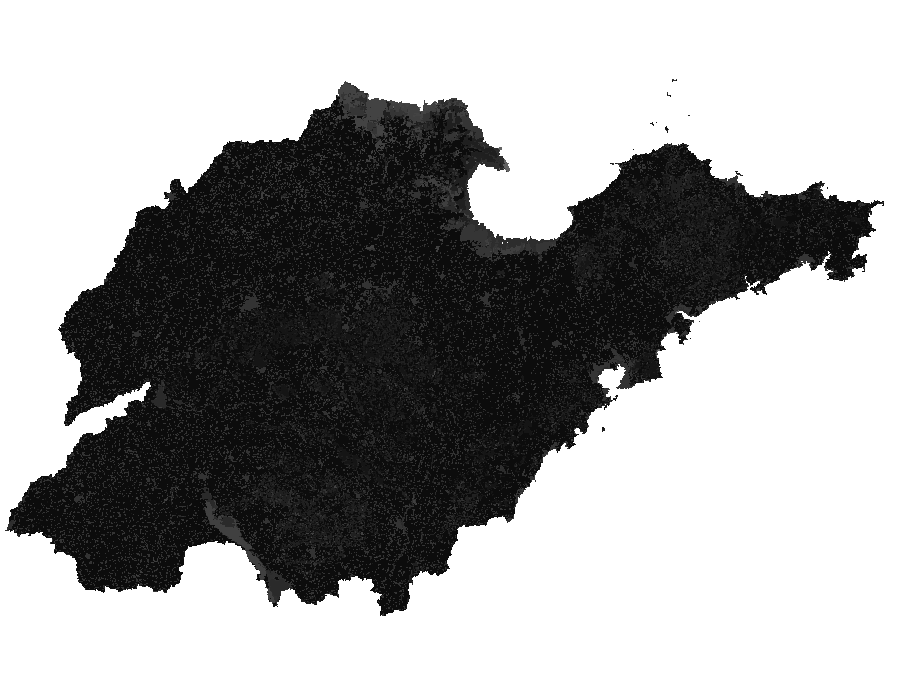

Supplement: Supplementary file 1 — Supplementary Information. [file 41598_2022_19493_MOESM1_ESM.zip › 41598_2022_19493_MOESM1_ESM/Data-tif/1990.tif]

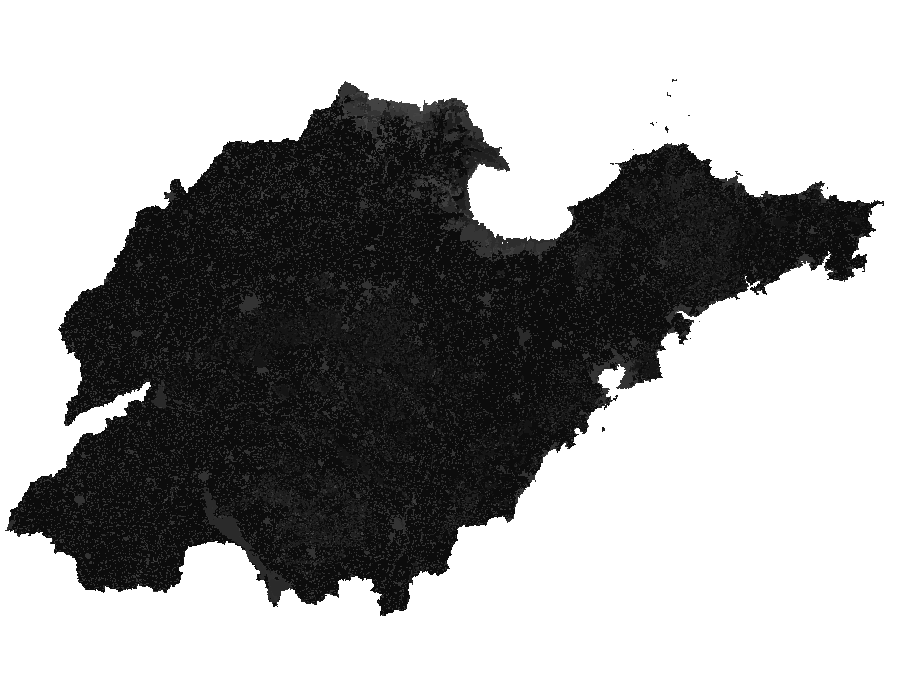

Supplement: Supplementary file 1 — Supplementary Information. [file 41598_2022_19493_MOESM1_ESM.zip › 41598_2022_19493_MOESM1_ESM/Data-tif/2000.tif]

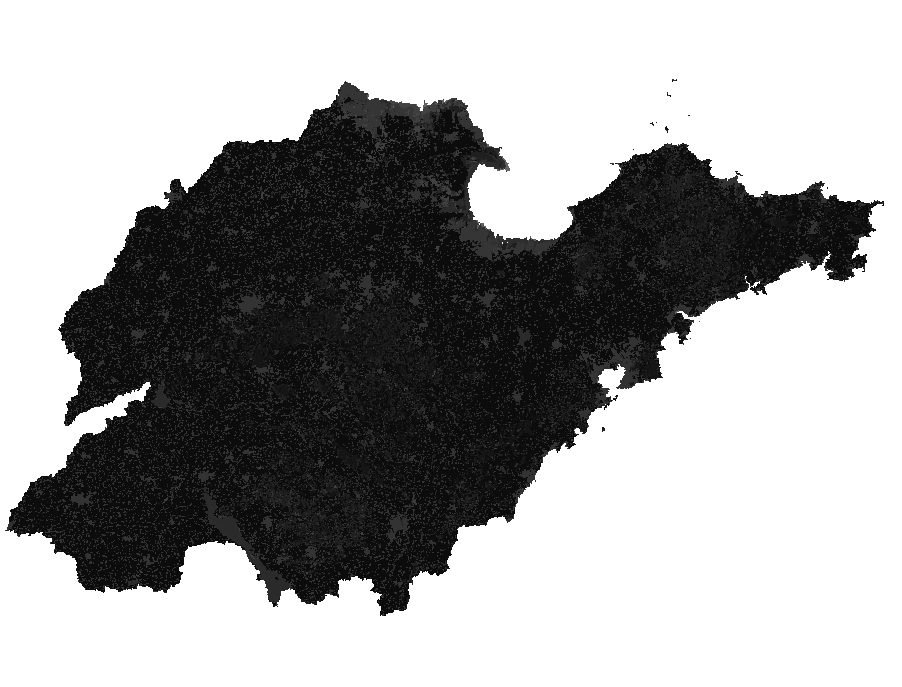

Supplement: Supplementary file 1 — Supplementary Information. [file 41598_2022_19493_MOESM1_ESM.zip › 41598_2022_19493_MOESM1_ESM/Data-tif/2010.tif]

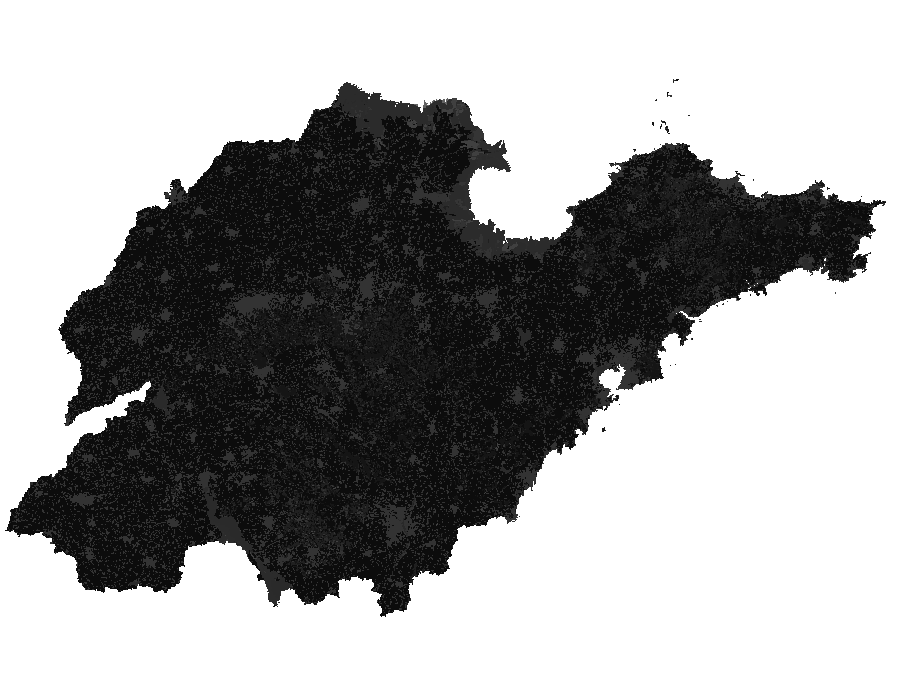

Supplement: Supplementary file 1 — Supplementary Information. [file 41598_2022_19493_MOESM1_ESM.zip › 41598_2022_19493_MOESM1_ESM/Data-tif/2020.tif]
